# Mini-Implant Rejection Rate in Teenage Patients Depending on Insertion Site: A Retrospective Study

**DOI:** 10.3390/jcm11185331

**Published:** 2022-09-10

**Authors:** Teodora Consuela Bungău, Luminița Ligia Vaida, Abel Emanuel Moca, Gabriela Ciavoi, Raluca Iurcov, Ioana Mihaela Romanul, Camelia Liana Buhaș

**Affiliations:** 1 Department of Dentistry, Faculty of Medicine and Pharmacy, University of Oradea, 10 Piața 1 Decembrie Street, 410073 Oradea, Romania; 2 Department of Morphological Disciplines, Faculty of Medicine and Pharmacy, University of Oradea, 410087 Oradea, Romania

**Keywords:** mini-implants, rejection rate, teenagers, insertion site

## Abstract

Mini-implants have undeniable advantages in Orthodontics. However, the use of mini-implants shows some limitations and disadvantages related to patient age, the quality of the bone tissue, the characteristics of the oral mucosa, implant site, the state of health of the organism and the quality of oral hygiene. The aim of this paper was to analyze the rejection rate of mini-implants in teenage patients, depending on their insertion site, and examine their stability up to three months after insertion. This retrospective study was conducted on dental charts belonging to patients aged between 12 and 17 years, from Oradea, Romania. The mini-implants were placed for various therapeutic reasons and were inserted in the following sites: buccal maxillary area, the infrazygomatic region, palatal area, buccal mandibular area and lingual area; they had a diameter of 1.6 mm (inter-radicular spaces) and of 2 mm (nonbearing tooth areas), and a length of 6–8 mm (mandible) or 8–10 mm (maxilla). The rejection rate was checked in the first month, second month, third month and after the third month from insertion. A total of 432 patients were included in the study, and they had a total of 573 mini-implants. Most implants were placed in the buccal region of the maxilla (27.7%), and most patients had one mini-implant placed (65.7%). The highest rejection rate was obtained in the first month (15.2%). The rejection rate between genders was similar. The mini-implants from the buccal mandibular region had a significantly higher rate of rejection in the first month (M1) in comparison to the mini-implants from the palatal region (24.4% vs. 8.3%). The mini-implants from the lingual region of the mandible had a significantly higher rate of rejection in the second month (M2) in comparison to the mini-implants from the infrazygomatic or the palatal region (10.5% vs. 0%/0%). Mini-implants are very useful for carrying out various orthodontic treatments, but their stability should be enhanced.

## 1. Introduction

In Orthodontics, anchorage is defined as the ability to resist undesired reactive tooth movements, and it can be obtained by using teeth, palate, head, neck and TADs (Temporary Anchorage Devices) [1]. TADs have become an increasingly used method for orthodontic treatments, and the skeletal anchorage they provide is fast replacing conventional anchorage [2]. In order to obtain an optimal anchorage, the ideal device should be easy to use, cheap, able to be loaded immediately, immobile, not require patient’s compliance, biocompatible and offer superior or comparable results to traditional anchorage systems [3]. Among the anchorage systems used in Orthodontics are osseointegrated implants, palatal implants, miniplates, miniscrew implants [4]. 

The miniscrew implants are also called mini-implants, this being the most used term in the orthodontic literature, and were developed as a mean to overcome the issue related to the large size of osseointegrated implants, which were used for orthodontic anchorage [5]. Mini-implants have undeniable advantages in Orthodontics [6]. They allowed for the reconsideration of anchorage principles and the biomechanics used in orthodontic treatment [7]. However, the use of mini-implants shows some limitations and disadvantages related to patient age [8], the quality of the bone tissue [9], the characteristics of the oral mucosa [10], implant site [11], the state of health of the organism [12] and the quality of patient oral hygiene [13].

Even though the failure rate shown in literature is approximately 10% [14], loose mini-implants during ongoing treatment, even for a minority of the cases, is a disadvantage. In addition to the factors listed above, mini-implant stability depends on the mechanical interlocking of threads and bony tissue and not on osseointegration. In order to obtain maximum efficiency, they should ideally remain immobile when orthodontic force is applied [15]. Literature shows that most miniscrew failure occurs in the first week after mini-implant insertion [16]. Moreover, loose mini-implants are often observed in teenagers [17]. This is likely to be related to active bone metabolism in growing children and to low maturation of the bone, including the maxillo-mandibular bone [18]. To the best of our knowledge, there are no recent studies that focus on investigating the mini-implant rejection rate in teenage patients, and research focusing on this age group was considered beneficial.

The aim of this paper was to analyze the distribution of the mini-implants according to the insertion site and gender in a sample of Romanian teenagers (12 to 17 years old). Another aim was to investigate the rejection rate of the mini-implants depending on the different insertion sites (buccal maxillary region, infrazygomatic region, palatal region, buccal mandibular region, lingual region) at one month, two months, three months and after three months from the insertion moment. Comparisons between boys and girls regarding the rejection rate of the mini-implants were also desired.

## 2. Materials and Methods

### 2.1. Ethical Considerations

The study was conducted in accordance with the 1964 Declaration of Helsinki and its later amendments and was approved by the Research Ethics Committee of the University of Oradea (IRB No. CEFMF/04 from 4 February 2022). All of the patients’ parents or legal guardians that took part in this study have given their permission to be included in this research.

### 2.2. Sample Selection

This retrospective study was conducted on dental charts belonging to teenage patients from Oradea, North-Western Romania. The dental charts were analyzed between 4 February 2022 and 10 February 2022 and were collected from a private orthodontic office in Oradea, Romania. We analyzed all charts that belonged to adolescent patients treated in the last 5 years (1 October 2016 to 1 October 2021). The last mini-implant that was analyzed was placed in 10 June 2021, so that it could be evaluated after more than three months as well.

We included in this study generally healthy patients aged between 12 and 17 years who received orthodontic treatment with fixed appliance and mini-implants and have previously given their permission to take part in the study. Approval of study participation was also obtained from patients’ parents or legal guardians.

Partially completed dental charts with missing information were excluded from the study. The excluded dental charts also belonged to patients from other countries or patients with local or general pathologies that could influence the maxillary bone structural integrity.

In order to avoid bias, all dental charts were initially examined by one author (T.C.B.) and were double checked by another author (A.E.M.). The inter-rater reliability was 93%. It showed a very good inter-rater agreement for the mini-implant failure, location of mini-implant, gender, age and purpose of mini-implants, respectively.

### 2.3. Mini-Implant Properties and Insertion Sites

Each patient that was included in this study had at least one mini-implant inserted and a maximum of 4 mini-implants, for various treatment reasons, such as:

- Correction of vertical dimension: molar intrusion in anterior open bite cases, lower molar intrusion in high angle patients, incisor intrusion in deep bite with excessive gingival display, and undesirable occlusal plane angulation;
- Correction of sagittal dimension: class II or class III biomechanics (intermaxillary elastic traction), anterior movement of the canine to substitute a missing lateral incisor (agenesis), distal or mesial movements of a single tooth or group of teeth for closing or opening up spaces, molar uprighting;
- Prosthetic cases which needed single tooth movement without a complete fixed appliance.

The following areas were used for mini-implant insertion:

- Buccal maxillary area: inter-radicular space between the maxillary second premolar and first molar; inter-radicular space between the upper lateral incisor and upper canine;
- The infrazygomatic region was analyzed separately considering the peculiarities of this area;
- Palatal area: midpalatal, paramedian area or inter-radicular space from the midline and up to and including the tuberosity space, depending on the purpose;
- Buccal mandibular area: interradicular space between lateral incisor and canine, up to mandibular first molar, mandibular second molar or the retromolar region;
- Lingual area: inter-radicular space from the lateral incisive to the retromolar area.

The diameter of the mini-implants was 1.6 mm for inter-radicular spaces and 2 mm for nonbearing tooth areas such as the midpalatal region or the zygomatic buttress. The length was 6–8 mm for the mandible and midpalatal area, and 8–10 mm for the maxilla or areas with thick mucosa (Table 1).

As far as the insertion technique is concerned, the self-drilling method was used for all of the patients and it was performed by the same practitioner. After placement, the initial stability of the mini-implant was checked to ensure there were no signs of mobility.

All of the mini-implants (except for the infrazygomatic region) were placed in attached gingiva. The infrazygomatic mini-implants were placed in alveolar mucosa. All of the mini-implants were immediately loaded (maximum of 24–48 h post-insertion) with a force between 100 and 150 g (1–1.5 N). The force was measured using a Teclock Push Pull Gauge (PPN 750-5).

The removal or loss of a mini-implant due to the onset of mobility between the first 24 h and up to six months after insertion was considered a failure. 

To serve the purpose of this study, a retrospective analysis was made to check the rejection rate of the mini-implants in the first-month post-insertion (M1), in the second month (M2), in the third month (M3) and after the third month. The distribution of the mini-implants according to the location and the rejection rate was also analyzed for each month. 

### 2.4. Statistical Analysis

All the data from the study was analyzed using IBM SPSS Statistics 25 (IBM, Chicago, IL, USA) and illustrated using Microsoft Office Excel/Word 2013 (Microsoft, Redmond, WA, USA). Quantitative variables were tested for normal distribution using the Shapiro–Wilk Test and were written as averages with standard deviations or medians with interquartile ranges. Qualitative variables were written as counts or percentages and were tested using Fisher’s Exact tests. Z-tests with Bonferroni correction were made to further detail the results obtained in the contingency tables. Odds ratio with 95% confidence intervals were used to illustrate the nature of association detected in contingency tables. Logistic regression models testing for goodness-of-fit and significance were used in prediction of the rejection rates in the first and second months. A *p* value of <0.05 was considered statistically significant.

## 3. Results

Initially, 485 patients were selected, but after applying the exclusion criteria, 432 patient dental charts were kept in this study. The 432 patients included in the study received a total of 573 mini-implants. 

Regarding the distribution of the patients related to gender, most patients were girls. Out of the 432 selected patients, 243 were girls (56.2%) and 189 were boys (43.8%). The average age was 14.31 ± 1.625 years, with a median of 14 years, and the age range was between 12 and 17 years. 

Data from Table 2 shows the distribution of the mini-implants according to their insertion site, with the most frequent insertion sites being the buccal part of the maxilla, the hard palate and the buccal part of the mandible. 

**Figure 1:**
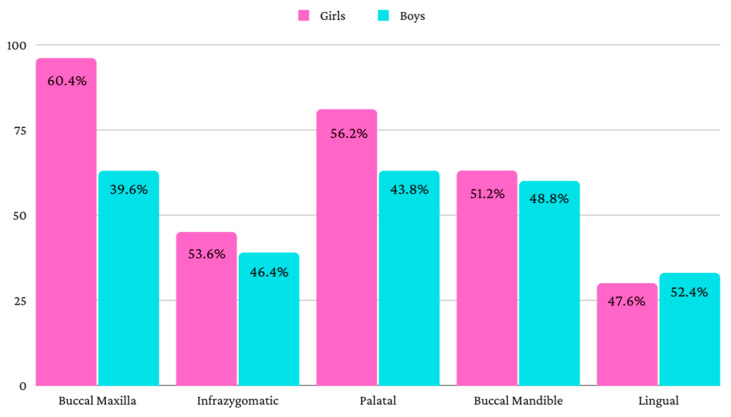
Distribution of the mini-implants for girls and for boys.

Figure 1 shows the distribution of the mini-implants according to the insertion site, separately for girls and for boys. The distribution was relatively similar for the infrazygomatic, buccal mandibular and lingual mini-implants. Larger differences were identified for buccal maxillary and palatal mini-implants.

Most of the analyzed patients had only one mini-implant (65.7%, *n* = 284) or two mini-implants (32.9%, *n* = 142). Only a small percentage of 0.70% of patients received three (*n* = 3) or four (*n* = 3) mini-implants.

**Figure 2:**
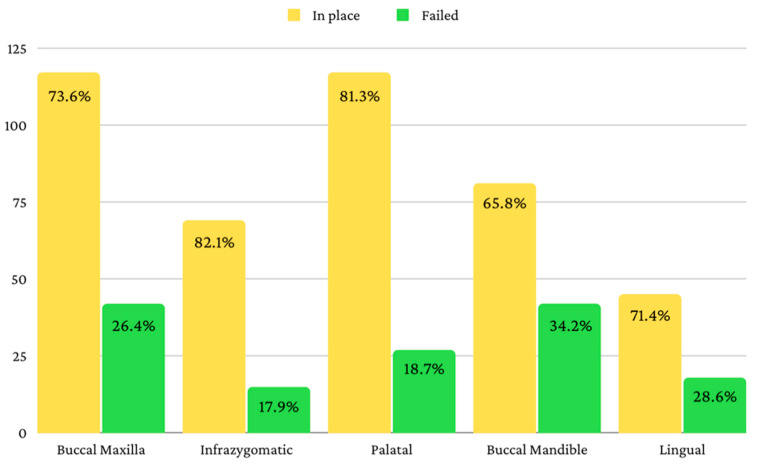
Mini-implants in place and rejected after more than three months.

The first month showed the highest rejection rate of 15.2% (*n* = 87). After that, 3.7% (*n* = 18) of the mini-implants were rejected in the second month, 1.9% (*n* = 9) of the mini-implants were rejected in the third month, and 6.5% (*n* = 30) of the mini-implants were rejected after the third month. Figure 2 shows the number of mini-implants that remained in place, and the number of mini-implants that failed according to the insertion site.

Furthermore, the distribution of the mini-implants according to the location and the rejection rate was analyzed for each month (Table 3).

The global rejection rate of the mini-implants in the first month was 15.2%, the observed differences between groups were statistically significant according to Fisher’s Exact Test (*p* = 0.002) and Z-tests with Bonferroni correction showed that the mini-implants from the buccal mandibular region had a significantly higher rate of rejection in the first month (M1) in comparison to the mini-implants from the palatal region (24.4% vs. 8.3%). Other differences between other regions were not statistically significant. 

In the second month (M2), the global rejection rate of the mini-implants was 3.7%, and the observed differences between groups were statistically significant according to Fisher’s Exact Test (*p* < 0.001) and Z-tests with Bonferroni correction showed that the mini-implants from the lingual region of the mandible had a significantly higher rate of rejection in the second month in comparison to the mini-implants from the infrazygomatic or the palatal region (10.5% vs. 0%/0%). Other differences between other regions were not statistically significant.

Because of the low rejection rates from the third month (M3) and after the third month, the observed differences between groups were not statistically significant according to Fisher’s Exact Test. 

A univariate logistic regression models used for the prediction of mini-implant rejection in the first month. Due to collinearity between implant length and implant region only univariate models were used. After testing for goodness-of-fit, the models had high significance (*p* < 0.001), showing the following results:

- In comparison to 10 mm implants, 6 mm implants had 2.283 higher odds of rejection in the first month (95% C.I.: 1.197–4.353) and 8 mm implants had 2.769 higher odds of rejection in the first month (95% C.I.: 1.601-4.788);
- In comparison to implants located in the vestibular maxilla, implants in the palatine region had 2.557 lower odds of rejection in the first month (95% C.I.: 1.254–5.208) and implants in the vestibular mandible had 1.779 higher odds of rejection in the first month (95% C.I.: 1.021–3.101).

Table 4 shows the distribution of mini-implants based on the patient’s gender and rejection in M1, M2, M3, and after M3. The observed differences between groups were not statistically significant according to Fisher’s Exact Test, the rejection rate being approximately similar between genders in M1, M3 and after M3. In the second month (M2) the rejection rate was significantly higher for girls than for boys (5.6% vs. 1.4%). As for the distribution of the mini-implants according to gender, insertion site and rejection rate (Table 5) the results obtained showed that most of the times the differences between gender were not statistically significant. However, the differences between genders were statistically significant for mini-implants inserted in the infrazygomatic region after M3, and for the mini-implants inserted in the lingual region at M1, M2, and after M3.

A univariate logistic regression models for the prediction of mini-implant rejection in the second month was performed. Due to collinearity between implant length and implant region only univariate models were used. After testing for goodness-of-fit and significance, only gender exhibited a significant prediction, showing that in comparison to male patients, implants in female patients had 4.25 higher odds of rejection in the second month (95% C.I.: 1.217–14.846).

The distribution of the mini-implants according to diameter and the rejection rate is shown in Table 6. The rejection rate was significantly more associated with 1.6 mm mini-implants in M1 and M2, and to 2 mm mini-implants after M3. The distribution of the mini-implants according to length and rejection rate is shown in Table 7. The rejection rate was significantly more associated with 6 or 8 mm mini-implants in M1 and M2.

## 4. Discussion

The indications for the use of mini-implants are numerous, and in many cases they can successfully replace other types of fixed orthodontic appliances, or sometimes they can be used as an alternative to orthognathic surgery [19]. They can also be used for the traction of transmigrated teeth [20]. Mini-implants do not require special surgical procedures, and are inserted easily. They can be inserted in various locations, do not require compliance from the patient and can be easily removed [21]. These advantages have led to an increase in the frequency of their use, but also to the extent of the indications of mini-implants [22].

The age of patients has proven to influence the success rate of mini-implants [23]. In this study, the group was composed of adolescent patients aged between 12 and 17 years. The age range was chosen because it is considered that growth spurts of the bone occur during infancy (1 to 4 years) and puberty (12 to 17 years) [24]. The lower limit of 12 years was also selected because some authors contraindicate the application of mini-implants in patients younger than 12 years old [25]. However, the chronological age of the patient is not always an indication of the skeletal development [26].

The mini-implants that were used in this study sample, had a diameter between 1.6 mm (inter-radicular spaces) and 2 mm (nonbearing tooth areas), and a length between 6–8 mm (mandible and midpalatal area) and 8–10 mm (maxilla and areas with thick mucosa). These dimensions fall within the limits recommended in the orthodontic literature. Suzuki et al. (2013) reached the conclusion that for a mini-implant with a diameter of 1.3 mm, the length of 5 mm (maxilla) and 6 mm (mandible) represent the minimum dimensions necessary for obtaining a good anchorage [27]. In general, mini-implants with a diameter smaller than 1.2 mm have higher chances of failure [28], and a short mini-implant placed in an insertion site with a thick mucosa has higher chances of being rejected, in these cases longer implants being recommended [29]. However, when it comes to skeletal anchorage, the length of the mini-implant is less important than the diameter of the mini-implant. The primary stability of a mini-implant is, also, better when the insertion angle is of 70 degrees [30]. The insertion technique used was the self-drilling technique because it reduces operative time, bone damage and patient discomfort, having a success rate similar to mini-implants inserted with the self-tapping technique [31]. The self-drilling technique, however, does not mean higher pressure. Applying excessive pressure while placing the mini-implant, can lead to mini-implant displacement, microfractures and alveolar bone enlargement [32].

In the investigated sample, most mini-implants were rejected in the first month of insertion (15.2%), and the fewest were rejected in the third month (1.9%). Alharbi et al. (2018) reported a rejection rate of 14.3% following the analysis of 11 studies with samples in which more than 100 implants were inserted [15], a result similar to that obtained in this study. Regarding adolescent patients, the same authors reported a failure rate of 8.6%, much lower than that obtained in this study [15]. Results closer to those obtained in this study sample were reported by Papageorgiou et al. (2012) who identified a failure rate of 12.6%, in patients under the age of 20 years [12]. Most mini-implants failed in the first month after insertion, a result consistent with those obtained by other authors who reported that most mini-implants fail in the first week after application [16].

In the first month after insertion, most mini-implants that failed were inserted in the buccal mandibular region (24.4% failure rate), and the fewest in the palatal region (8.3% failure rate). Failure rates of mini-implants placed in the buccal mandibular region of 7.2% were also reported, but those mini-implants were placed in the buccal mandibular shelf [33]. Higher failure rates, of 19.3%, have also been reported [33]. Some authors reported a failure rate of 39.1% of mini-implants placed in the buccal region of the mandible, and of 23.1% of mini-implants placed in the buccal region of the maxilla, at 12 months [34]. Regarding the failure rate of palatal mini-implants, this was similar to other studies that reported a failure rate of 6.1% [35] or 8.5% [34]. A single study reported a failure rate of 16.7%, much higher than that reported in this study [36].

Infrazygomatic placed mini-implants had a failure rate of 10.7% in the first month, and 0% in the second and third month. Uribe et al. (2015) obtained a failure rate of 21.8% of mini-implants placed in the infrazygomatic region [37], while Liou et al. (2004) reported a 100% success rate of mini-implants placed in the infrazygomatic region [38]. 

The failure rates of the mini-implants depending on the gender of the patients were similar between boys and girls. In the first month, the failure rate was 14.3% for girls and 16.3% for boys and decreased in the second and third month. Some authors have reported bigger differences between genders, regarding the failure rate of mini-implants [39], but others have reported similar failure rates between the two genders [40].

Mini-implants were reported to have high success rates (≥90%), which depended on device-related factors, patient-related factors, procedure-related factors, and orthodontic treatment-related factors [41,42]. Mini-implants are useful because they lead to a more efficient orthopedic development in patients with active growth [43]. The study of mini-implants must continue in order to discover the most effective application methods, the best insertion sites, but also the most suitable design, considering that their stability is affected by a variety of factors [44].

This study has some limitations. First of all, it is a retrospective study, so the accuracy regarding the age of the patients, the place of insertion and the dimensions of the mini-implants could have been erroneously recorded. Secondly, the group is limited to the patients of a single orthodontic office, so factors related to the insertion method, preference for certain mini-implants and other aspects related to therapeutic decisions may be specific to the operator.

## 5. Conclusions

In this study sample, the highest failure rate occurred in the first month, and the lowest in the third month. In the first month, mini-implants placed in the buccal mandibular region had a significantly higher rate of rejection in comparison to mini-implants placed in the palatal region, while in the second month mini-implants from the lingual region of the mandible had a significantly higher rate of rejection in comparison to the mini-implants from the infrazygomatic or the palatal region. Patients’ gender did not influence the failure rate, with this being similar between girls and boys. 

## Figures and Tables

**Table 1:** Diameter and length used depending on the insertion site.

Location	Diameter (mm)	Length (mm)
Buccal maxilla	1.6	8–10
Infrazygomatic	2	8–10
Palatal	2	8–10
Buccal mandible	1.6	6–8
Lingual	1.6	6–8

**Table 2:** Distribution of the mini-implants according to the insertion site.

Location	No.	Percentage
Buccal maxilla	159	27.7%
Infrazygomatic	84	14.7%
Palatal	144	25.1%
Buccal mandible	123	21.5%
Lingual	63	11%

No., number.

**Table 3:** Distribution of the mini-implants according to the location and the rejection rate in M1, M2, M3 and after M3.

Location (No. /%)	Buccal Maxilla	Infrazygomatic	Palatal	Buccal Mandible	Lingual	*p* *
**M1**						
In place	129 (81.1%)	75 (89.3%)	132 (91.7%)	93 (75.6%)	57 (90.5%)	0.002
Failed	30 (18.9%)	9 (10.7%)	12 (8.3%)	30 (24.4%)	6 (9.5%)	
M2						
In place	123 (95.3%)	75 (100%)	132 (100%)	87 (93.5%)	51 (89.5%)	<0.001
Failed	6 (4.7%)	0 (0%)	0 (0%)	6 (6.5%)	6 (10.5%)	
**M3**						
In place	120 (97.6%)	75 (100%)	129 (97.7%)	84 (96.6%)	51 (100%)	0.489
Failed	3 (2.4%)	0 (0%)	3 (2.3%)	3 (3.4%)	0 (0%)	
**After M3**						
In place	117 (97.5%)	69 (92%)	117 (90.7%)	81 (96.4%)	45 (88.2%)	0.056
Failed	3 (2.5%)	6 (8%)	12 (9.3%)	3 (3.6%)	6 (11.8%)	

No., number; %, percentage; * Fisher’s Exact Test.

**Table 4:** Distribution of the mini-implants according to gender and the rejection rate in M1, M2, M3 and after M3.

Gender/Rejection	Girls		Boys		*p* *
	No.	%	No.	%	
**M1**					
In place	270	85.7%	216	83.7%	0.559
Failed	45	14.3%	42	16.3%	
**M2**					
In place	255	94.4%	213	98.6%	0.016
Failed	15	5.6%	3	1.4%	
**M3**					
In place	249	97.6%	210	98.6%	0.520
Failed	6	2.4%	3	1.4%	
**After M3**					
In place	249	97.6%	210	98.6%	0.520
Failed	6	2.4%	3	1.4%	

No., number; %, percentage; * Fisher’s Exact Test.

**Table 5:** Distribution of the mini-implants according to gender, insertion site and rejection rate in M1, M2, M3 and after M3.

Location (No./%)	Buccal Maxilla		Infrazygomatic		Palatal		Buccal Mandible		Lingual	
	G	B	G	B	G	B	G	B	G	B
**M1**										
In place	81 (84.4%)	48 (76.2%)	39 (86.7%)	36 (92.3%)	75 (92.6%)	57 (90.5%)	42 (66.7%)	45 (75%)	30 (100%)	27 (81.8%)
Failed	15 (15.6%)	15 (23.8%)	6 (13.3%)	3 (7.7%)	6 (7.4%)	6 (9.5%)	21 (33.3%)	15 (25%)	0 (0%)	6 (18.2%)
*p* *	0.218		0.494		0.764		0.329		0.025	
**M2**										
In place	90 (93.8%)	63 (100%)	45 (100%)	39 (100%)	81 (100%)	63 (100%)	60 (95.2%)	57 (95%)	24 (80%)	33 (100%)
Failed	6 (6.2%)	0 (0%)	0 (0%)	0 (0%)	0 (0%)	0 (0%)	3 (4.8%)	3 (5%)	6 (20%)	0 (0%)
*p* *	0.082		-		-		1.000		0.009	
**M3**										
In place	96 (100%)	60 (95.2%)	45 (100%)	39 (100%)	78 (96.3%)	63 (100%)	60 (95.2%)	60 (100%)	30 (100%)	33 (100%)
Failed	0 (0%)	3 (4.8%)	0 (0%)	0 (0%)	3 (3.7%)	0 (0%)	3 (4.8%)	0 (0%)	0 (0%)	0 (0%)
*p* *	0.060		-		0.257		0.244		-	
**After M3**										
In place	96 (100%)	60 (95.2%)	45 (100%)	33 (84.6%)	75 (92.6%)	57 (90.5%)	63 (100%)	57 (95%)	24 (80%)	33 (100%)
Failed	0 (0%)	3 ( 4.8%)	0 (0%)	6 (15.4%)	6 (7.4%)	6 (9.5%)	0 (0%)	3 (5%)	6 (20%)	0 (0%)
*p* *	0.060		0.008		0.764		0.113		0.009	

No., number; %, percentage; * Fisher’s Exact Test; G, girls; B, boys.

**Table 6:** Distribution of the mini-implants according to implant diameter and the rejection rate in M1, M2, M3 and after M3.

Diameter (No./%)	1.6 mm	2 mm	*p* *
**M1**			
In place	273 (79.1%)	207 (90.8%)	<0.001
Failed	72 (20.9%)	21 (9.2%)	
**M2**			
In place	327 (94.8%)	228 (100%)	<0.001
Failed	18 (5.2%)	0 (0%)	
**M3**			
In place	339 (98.3%)	225 (98.7%)	1.000
Failed	6 (1.7%)	3 (1.3%)	
**After M3**			
In place	333 (96.5%)	210 (92.1%)	0.033
Failed	12 (3.5%)	18 (7.9%)	

No., number; %, percentage; * Fisher’s Exact Test.

**Table 7:** Distribution of the mini-implants according to implant length and the rejection rate in M1, M2, M3 and after M3.

Length (No./%)	6 mm	8 mm	10 mm	*p* *
**M1**				
In place	95 (81.2%)	178 (78.1%)	207 (90.8%)	0.001
Failed	22 (18.8%)	50 (21.9%)	21 (9.2%)	
**M2**				
In place	108 (92.3%)	219 (96.1%)	228 (100%)	<0.001
Failed	9 (7.7%)	9 (3.9%)	0 (0%)	
**M3**				
In place	116 (99.1%)	223 (97.8%)	225 (98.7%)	0.750
Failed	1 (0.9%)	5 (2.2%)	3 (1.3%)	
**After M3**				
In place	112 (95.7%)	221 (96.9%)	210 (92.1%)	0.064
Failed	5 (4.3%)	7 (3.1%)	18 (7.9%)	

No., number; %, percentage; * Fisher’s Exact Test.

## Data Availability

The data presented in this study are available on request from the corresponding authors. The data are not publicly available due to privacy reasons.

## References

[1] Feldmann I., Bondemark L. (2006). Orthodontic anchorage: A systematic review. Angle Orthod..

[2] Leo M., Cerroni L., Pasquantonio G., Condò S.G., Condò R. (2016). Temporary anchorage devices (TADs) in orthodontics: Review of the factors that influence the clinical success rate of the mini-implants. Clin. Ter..

[3] Cope J.B. (2005). Temporary anchorage devices in orthodontics: A paradigm shift. Semin. Orthod..

[4] McGuire M.K., Scheyer E.T., Gallerano R.L. (2006). Temporary anchorage devices for tooth movement: A review and case reports. J. Periodontol..

[5] Reynders R., Ronchi L., Bipat S. (2009). Mini-implants in orthodontics: A systematic review of the literature. Am. J. Orthod. Dentofacial Orthop..

[6] Elias C.N., de Oliveira Ruellas A.C., Fernandes D.J. (2012). Orthodontic implants: Concepts for the orthodontic practitioner. Int. J. Dent..

[7] Cousley R.R.J. (2015). Mini-implants in contemporary orthodontics part 2: Clinical applications and optimal biomechanics. Orthod. Update.

[8] Chen Y.J., Chang H.H., Huang C.Y., Hung H.C., Lai E.H., Yao C.C. (2007). A retrospective analysis of the failure rate of three different orthodontic skeletal anchorage systems. Clin. Oral Implant. Res..

[9] Tseng Y.C., Hsieh C.H., Chen C.H., Shen Y.S., Huang I.Y., Chen C.M. (2006). The application of mini-implants for orthodontic anchorage. Int. J. Oral Maxillofac. Surg..

[10] Kim H.J., Yun H.S., Park H.D., Kim D.H., Park Y.C. (2006). Soft-tissue and cortical-bone thickness at orthodontic implant sites. Am. J. Orthod. Dentofac. Orthop..

[11] Wu T.Y., Kuang S.H., Wu C.H. (2009). Factors associated with the stability of mini-implants for orthodontic anchorage: A study of 414 samples in Taiwan. J. Oral Maxillofac. Surg..

[12] Papageorgiou S.N., Zogakis I.P., Papadopoulos M.A. (2012). Failure rates and associated risk factors of orthodontic miniscrew implants: A meta-analysis. Am. J. Orthod. Dentofac. Orthop..

[13] Kravitz N.D., Kusnoto B. (2007). Risks and complications of orthodontic miniscrews. Am. J. Orthod. Dentofac. Orthop..

[14] Park H.S., Jeong S.H., Kwon O.W. (2006). Factors affecting the clinical success of screw implants used as orthodontic anchorage. Am. J. Orthod. Dentofac. Orthop..

[15] Alharbi F., Almuzian M., Bearn D. (2018). Miniscrews failure rate in orthodontics: Systematic review and meta-analysis. Eur. J. Orthod..

[16] Kuroda S., Tanaka E. (2014). Risks and complications of miniscrew anchorage in clinical orthodontics. Jpn. Dent. Sci..

[17] Park H.S., Lee S.K., Kwon O.W. (2005). Group distal movement of teeth using microscrew implant anchorage. Angle Orthod..

[18] Motoyoshi M., Matsuoka M., Shimizu N. (2007). Application of orthodontic mini-implants in adolescents. Int. J. Oral Maxillofac. Surg..

[19] Singh K., Kumar D., Jaiswal R.K., Bansal A. (2010). Temporary anchorage devices—Mini-implants. Natl. J. Maxillofac. Surg..

[20] Vaida L., Todor B.I., Corega C., Băciuţ M., Băciuţ G. (2014). A rare case of canine anomaly—A possible algorithm for treating it. Rom. J. Morphol. Embryol..

[21] Papadopoulos M.A., Tarawneh F. (2007). The use of miniscrew implants for temporary skeletal anchorage in orthodontics: A comprehensive review. Oral Surg. Oral Med. Oral Pathol. Oral Radiol. Endod..

[22] Mizrahi E. (2016). The Use of Miniscrews in Orthodontics: A Review of Selected Clinical Applications. Prim. Dent. J..

[23] Jing Z., Wu Y., Jiang W., Zhao L., Jing D., Zhang N., Cao X., Xu Z., Zhao Z. (2016). Factors Affecting the Clinical Success Rate of Miniscrew Implants for Orthodontic Treatment. Int. J. Oral Maxillofac. Implant..

[24] Ohta H. (2019). Growth spurts of the bone from infancy to puberty. Clin. Calcium..

[25] Nausheer A., Rithika J., Abrar Y.A., Ranjan R.B.K. (2020). Temporary anchorage devices in orthodontics: A review. IJODR.

[26] Moca A.E., Vaida L.L., Moca R.T., Țuțuianu A.V., Bochiș C.F., Bochiș S.A., Iovanovici D.C., Negruțiu B.M. (2021). Chronological Age in Different Bone Development Stages: A Retrospective Comparative Study. Children.

[27] Suzuki M., Deguchi T., Watanabe H., Seiryu M., Iikubo M., Sasano T., Fujiyama K., Takano-Yamamoto T. (2013). Evaluation of optimal length and insertion torque for miniscrews. Am. J. Orthod. Dentofacial Orthop..

[28] Stanford N. (2011). Mini-screws success rates sufficient for orthodontic treatment. Evid. Based Dent..

[29] Topouzelis N., Tsaousoglou P. (2012). Clinical factors correlated with the success rate of miniscrews in orthodontic treatment. Int. J. Oral Sci..

[30] Tatli U., Alraawi M., Toroğlu M.S. (2019). Effects of size and insertion angle of orthodontic mini-implants on skeletal anchorage. Am. J. Orthod. Dentofac. Orthop..

[31] Yi J., Ge M., Li M., Li C., Li Y., Li X., Zhao Z. (2017). Comparison of the success rate between self-drilling and self-tapping miniscrews: A systematic review and meta-analysis. Eur. J. Orthod..

[32] Romano F.L., Consolaro A. (2015). Why are mini-implants lost: The value of the implantation technique!. Dent. Press J. Orthod..

[33] Chang C., Liu S.S., Roberts W.E. (2015). Primary failure rate for 1680 extra-alveolar mandibular buccal shelf mini-screws placed in movable mucosa or attached gingiva. Angle Orthod..

[34] Arqub S.A., Gandhi V., Mehta S., Palo L., Upadhyay M., Yadav S. (2021). Survival estimates and risk factors for failure of palatal and buccal mini-implants. Angle Orthod..

[35] Kakali L., Alharbi M., Pandis N., Gkantidis N., Kloukos D. (2019). Success of palatal implants or mini-screws placed median or paramedian for the reinforcement of anchorage during orthodontic treatment: A systematic review. Eur. J. Orthod..

[36] Takaki T., Tamura N., Yamamoto M., Takano N., Shibahara T., Yasumura T., Nishii Y., Sueishi K. (2010). Clinical study of temporary anchorage devices for orthodontic treatment--stability of micro/mini-screws and mini-plates: Experience with 455 cases. Bull. Tokyo Dent. Coll..

[37] Uribe F., Mehr R., Mathur A., Janakiraman N., Allareddy V. (2015). Failure rates of mini-implants placed in the infrazygomatic region. Prog. Orthod..

[38] Liou E.J., Pai B.C., Lin J.C. (2004). Do miniscrews remain stationary under orthodontic forces?. Am. J. Orthod. Dentofac. Orthop..

[39] Manni A., Cozzani M., Tamborrino F., De Rinaldis S., Menini A. (2011). Factors influencing the stability of miniscrews. A retrospective study on 300 miniscrews. Eur. J. Orthod..

[40] Lim H.J., Choi Y.J., Evans C.A., Hwang H.S. (2011). Predictors of initial stability of orthodontic miniscrew implants. Eur. J. Orthod..

[41] Ramírez-Ossa D.M., Escobar-Correa N., Ramírez-Bustamante M.A., Agudelo-Suárez A.A. (2020). An Umbrella Review of the Effectiveness of Temporary Anchorage Devices and the Factors That Contribute to Their Success or Failure. J. Evid. Based Dent. Pract..

[42] Casaña-Ruiz M.D., Bellot-Arcís C., Paredes-Gallardo V., García-Sanz V., Almerich-Silla J.M., Montiel-Company J.M. (2020). Risk factors for orthodontic mini-implants in skeletal anchorage biological stability: A systematic literature review and meta-analysis. Sci. Rep..

[43] Bucur S.-M., Vaida L.L., Olteanu C.D., Checchi V. (2021). A Brief Review on Micro-Implants and Their Use in Orthodontics and Dentofacial Orthopaedics. Appl. Sci..

[44] Wilmes B., Rademacher C., Olthoff G., Drescher D. (2006). Parameters affecting primary stability of orthodontic mini-implants. J. Orofac. Orthop..

